# Resource allocation is determined by both parents and offspring in a burying beetle

**DOI:** 10.1111/jeb.13692

**Published:** 2020-09-14

**Authors:** Maarit I. Mäenpää, Per T. Smiseth

**Affiliations:** ^1^ Ashworth Laboratories Institute of Evolutionary Biology University of Edinburgh Edinburgh UK

**Keywords:** begging, burying beetle, control over parental care, nutritional independence, parent–offspring conflict, provisioning

## Abstract

Parents and offspring have different optima for the level of parental resource allocation and the timing of nutritional independence. Theoretical models assume that either parents or offspring control the allocation of resources within a brood; however, control may also be mutual. Here, we investigate whether the resolution of parent–offspring conflict is biased towards cues from either the parents' or the offspring's behaviour, or whether the conflict is under mutual control. Importantly, we considered potential shifts in the power continuum over the entire period of juvenile dependency. The burying beetle *Nicrophorus vespilloides* parents provision food for the larvae, and the larvae solicit food from their parents with conspicuous begging displays. Both parental and larval behaviours change as larvae age. We repeatedly manipulated the age of the brood females care for, thereby creating mismatch between the age of the foster brood and expected age of the brood from the female parent's perspective, over the period of dependency in juvenile development. We found that females adjusted the total amount of provisioning based on the actual age of the brood. However, both the parent and the offspring influenced the levels of food provisioning, which followed neither the expected age of the brood from the parent's perspective nor offspring age. Our results suggest that there is mutual control over parental care, thus contradicting the dichotomous view of control over parental care. We suggest that the mutual influence of both parents and the offspring should be taken into account in development of future theory, as well as empirical studies.

## Graphical abstract

Control over resource allocation shifts from parents to the offspring in the burying beetle *Nicrophorus vespilloides* over the course of the period of dependency. Female parents adjust the total amount of provisioning based on age of the offspring they are caring for regardless of their own perception of brood age, but not to match offspring need fully. Our results suggest that there is mutual control over parental resource allocation.

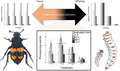

## 1 INTRODUCTION

Conflicts over allocation of resources and the timing of nutritional independence between caring parents and their dependent offspring are common, reflecting that offspring are under selection to extract more care from the parent than the parent is selected to provide (Godfray, 1995b; Parker & Macnair, 1979; Trivers, 1974). In many taxa, including mammals, birds, amphibia and insects, the resolution of this conflict is mediated through offspring begging displays that are used by offspring to obtain food from the parents (Kilner & Johnstone, 1997; Smiseth, Wright, & Kölliker, 2008; Trivers, 1974). Theoretical models for the evolutionary resolution of this conflict via begging signals fall into two different categories based on their assumptions about who controls resource allocation: Honest signalling models assume that the allocation of resources is controlled by parents, which choose which offspring to feed from a pool of competing signals (Godfray, 1991). Alternatively, scramble competition models assume that resource allocation is controlled by the offspring, as parents passively feed the offspring that present the greatest stimulus (Parker, Royle, & Hartley, 2002). Behavioural plasticity may play important role in determining who controls resource allocation as each party might seek to divert the other away from its optimum. Here we use the term “control” to refer to whether the outcome of the conflict between parents and offspring is shifted towards either party. For example, assuming that offspring are under selection to demand more resources than parents are prepared to provide (Trivers, 1974), an increase in provisioning to offspring would represent a shift towards greater offspring control. Owing to this, behavioural plasticity that shifts the amount of care provided by parents towards their offspring could reveal whether the parents or the offspring have the upper hand in the conflict, and thus which model is most appropriate for the resolution of parent–offspring conflict. While control over resource allocation varies based on the taxa and the environmental factors (Royle, Hartley, & Parker, 2002), few studies have explored how these two different models can operate over the duration of the period of dependency within the same broods.

The benefits and costs of offspring begging and parental provisioning of resources are likely to change over time as offspring age (Hinde, Johnstone, & Kilner, 2010; Royle et al., 2002). These changes lead to age‐dependent coadaptation in parental provisioning and offspring begging, which are expressed as matches between the behaviour of parents and offspring at a given offspring age (Gómez & Kölliker, 2013). In general, the offspring perform less well when the age‐dependent coadaptation is disrupted (Gómez & Kölliker, 2013; Hinde et al., 2010; Rehling et al., 2012). However, parents often adjust their behaviour to offspring need and may thus compensate for the effects of any mismatch in the age that the parent expects the offspring to be and the offspring's real age (Bruce, 1958; Djerdali, Tortosa, & Doumandji, 2008; Kight, 1997; Price, 1998; Rehling et al., 2012): Parents can express plasticity in their caring behaviour either by extending the period of parental care (e.g. Bruce, 1958; Kight, 1997; Rehling et al., 2012, but see Rehling & Trillmich, 2007), or by providing higher levels of care (e.g. Djerdali et al., 2008; Price, 1998, but see Riou, Chastel, & Hamer, 2012). Offspring begging can also be plastic, as offspring can adjust this behaviour to match their past experience of provisioning levels, either via innate mechanisms (Mäenpää, Andrews, Collette, Leigh, & Smiseth, 2015) or through learning (Kedar, Rodríguez‐Gironés, Yedvab, Winkler, & Lotem, 2000), or because of genetic correlations between parental and offspring traits (Agrawal, Brodie, & Brown, 2001). As both parents and offspring behaviours are plastic, both have the potential to control resource allocation by inducing a change in the other party's behaviour. The propensity for this plasticity may, however, change as the costs and benefits of care change with offspring age. Thus, to understand the dynamics in control over resource allocation requires an experimental design that induces repeated mismatches of the age‐dependent coadaptation between parents and offspring throughout the period of dependency.

The burying beetle, *Nicrophorus vespilloides*, is an excellent study system for investigating offspring begging and parental food provisioning, as the adult beetles exhibit elaborate parental care for their larvae, which they raise on carcasses of small vertebrates (Eggert & Müller, 1997; Scott, 1998). Although the larvae are capable of feeding from the carcass on their own (Eggert, Reinking, & Müller, 1998), they also beg for food from their parents by touching the adult with their legs, after which the parents regurgitate pre‐digested carrion for them to feed on (Smiseth, Darwell, & Moore, 2003; Smiseth & Moore, 2002). Both parents are involved in parental care, although usually only the females stay with the brood until the larvae disperse from the carcass into the soil to pupate (Scott, 1998). Larval begging behaviour changes over time as the larvae become more proficient in self‐feeding (Smiseth et al., 2003). The behaviour peaks at 24 hr after hatching, and declines thereafter, until approximately 72 hr after hatching, which marks the point of transitioning to nutritional independence (Smiseth et al., 2003). Notably, the larvae reach nutritional independence before they cease interacting with their parent completely (Smiseth et al., 2003), giving rise to potential for the parent or the offspring to prolong the period of parental care, unlike in species where the offspring stop interacting with the parent at the time of nutritional independence. Furthermore, parental provisioning of food to larvae also changes over time as the larvae age (Smiseth et al., 2003). This behaviour also peaks at 24 hr after hatching and parents normally cease provisioning food approximately 72 hr after hatching. Similar pattern in the behaviours of both the parents and the offspring indicate strong behavioural matches between the two.

**FIGURE 1:**
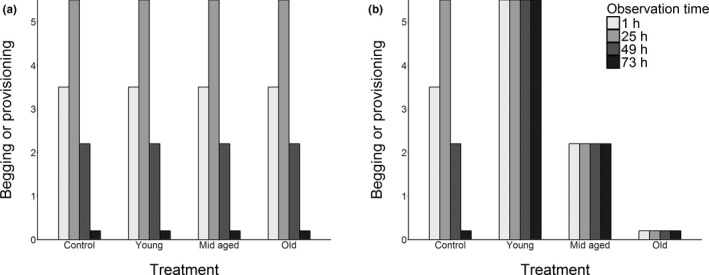
Two hypothetical extremes for the expected patterns of begging and provisioning over time based on assumptions of different situations: (a) Full parental control, where parents exhibit no plasticity in level of care or its duration, but offspring respond to the behaviour of the parent. No changes from the pattern of control treatment are expected across all treatments. (b) Full offspring control, where offspring exhibit no plasticity in the level of begging, but parents fully respond to offspring begging behaviour. The levels of behaviours exhibited do not change over time, but stay at the level as indicated by offspring age throughout the experiment. Patterns are drawn after empirical evidence of Smiseth et al. (2003)

In this experiment, we mismatched age‐specific coadaptation between offspring begging and parental provisioning in order to investigate whether the parents or the larvae were in control of food allocation and transition to nutritional independence in *N. vespilloides*. To this end, we conducted a cross‐fostering experiment where we provided parents with a new foster brood every 24 hr. We created mismatch between the age that the parents expected the brood to be and actual age of the offspring through repeated manipulations of the age of the brood that a female was caring for throughout offspring development. We imply no cognitive mechanisms by the use of the word “expected”, as its meaning hereafter simply refers to the time elapsed since larval hatching from the parents perspective. In our experimental treatments, we provided parents with foster broods such that the broods remained at the early, mid, or late stage of juvenile development, respectively. Meanwhile, in the control treatment, we provided parents with foster broods in a way that reflected the natural age of the parent's biological brood. We monitored subsequent effects on patterns of food provisioning, begging, and overall parental care in these treatments. We predict two hypothetical extremes that reflect whether the allocation of resources or the transition to nutritional independence is under parental or offspring control: (a) Parents have full control over the amount of care given and exhibit no plasticity in level of care or its duration through the entire period of dependency, but offspring fully respond to the behaviour of the parent (Figure 1a); (b) Offspring have full control over the amount of care given, and exhibit no plasticity in the level of begging, while parents fully respond to the begging behaviour of the offspring (Figure 1b). Additionally, to explore the consequences of the potential changes in the amount of care given, we also explored whether mismatches in age‐dependent coadaptation would change the timing of larval dispersal, which marks the time of complete separation from the parents, and thus whether the mismatch would have an effect on offspring development. Our experiment thus provides a powerful exploration of the amount of plasticity in both parental and offspring behaviours throughout offspring development, providing us with insights into how the power struggle between the two is resolved over time.

## 2 MATERIALS AND METHODS

### 2.1 Origin and husbandry of the beetles

The beetles used in the experiment were derived from a large, outbred laboratory population originating from wild‐caught beetles trapped in Corstorphine Hill and Craiglockhart Hill (Edinburgh, UK), Kennall Vale (Cornwall, UK), and Madingley Wood (Cambridge, UK). Each beetle in the laboratory population was kept individually in transparent plastic containers (7 × 12 × 6 cm) under constant light at 20°C, and fed small pieces of organic beef twice a week.

### 2.2 Experimental design

We randomly selected pairs of nonsibling virgin male and female beetles to be mated. The pairs were moved to a transparent container (12 × 18 × 6 cm) filled with 2 cm of moist soil and a previously frozen mouse carcass (range 20–25 g, supplied by Livefoods Direct Ltd). Male care is highly variable and has no detectable effects on larval growth or survival under laboratory conditions (Eggert et al., 1998; Smiseth, Dawson, Varley, & Moore, 2005), and therefore we only used female parents in this experiment. We removed the male 60 hr after pairing, before the larvae started hatching. Concurrently, we moved the female and the carcass into a new container filled with soil in order to separate the eggs from the breeding female. We did this to ensure that females had no larvae of their own at the time that we provided them with foster broods of known age. The egg boxes were checked five times a day for hatching.

We created mismatch between the actual age of the foster brood that the parent cared for and the age the parent expected it to be by repeated cross‐fostering throughout larval development. Manipulations of offspring age were conducted by swapping the brood that an experimental female was caring for, with another experimental brood of a known age every 24 hr for the approximate duration of larval dependency (i.e. first 72 hr after hatching). In order to achieve this, we needed a supply of larvae of known ages throughout the experiment. To this end, we generated mixed maternity donor broods consisting of larvae of an appropriate age that were cared for by a non‐experimental female foster parent until used in the experiment. For each experimental female, we set up two to three donor broods daily, to ensure that we had access to an excess number of larvae. The donor broods were created at a time corresponding to the time at which we observed the behaviour of the experimental female; every 24 (±15 min) hours over four consecutive days. We picked 15–25 newly hatched mixed maternity larvae for each donor brood, and moved these broods into a container with a female parent and a mouse carcass. We only used females whose own eggs had started hatching to avoid filial cannibalism (Müller & Eggert, 1990). We used mixed maternity broods in order to exclude potential effects of shared genetics between family members from the behaviour traits we aimed to explore. All broods were therefore foster broods of mixed origin, and no parent cared for its biological brood at any point during the experiment. The donor broods were used to create experimental broods of 10 larvae at subsequent stages of the experiment. In line with established protocols (Smiseth et al., 2003), we regard the age of a larvae as the time elapsed since it had access to food and thus could begin to grow.

We had four experimental treatments in this experiment, all of which followed the same general procedures: At the beginning of the experiment, an experimental female was given a brood of 10 larvae of a known age. An hour later, we conducted a behavioural observation on the first brood, after which the brood was removed and replaced by another brood created from the appropriate batch of donor broods. All larvae taken away after the observations were returned into the pool of donors to be used to generate experimental broods later in the experiment for experimental females in other treatments. The observations for a given female were then repeated three times at 24‐hr intervals, with the larvae being swapped after each observation, aside from the last, after which the female was allowed to raise the larvae until they dispersed from the carcass. When all larvae had moved from the carcass to the soil around it, we removed the female and ended the experiment. We then calculated the age of the larvae at dispersal (in days) by adding the number of days it took for them to disperse to their age at the end of the last observation.

Our experimental treatments differed in the age of the brood that was used to replace the previous brood. (a) In the control treatment, the initial broods were set up using newly hatched larvae, observed as they were 1‐hr old, and later the broods were always replaced by broods consisting of larvae of the same age as the ones that were taken away. This was done in order to control for any potential effects of swapping the broods. The broods were therefore observed at the age of 1, 25, 49, and 73 hr, and the actual age of the brood always matched the parental expectations of larval age. In the rest of the treatments, the expectations of the parent and the actual age of the brood were mismatched by manipulating the ages of the broods as follows: (b) In the young treatment, the larvae remained at the early stage of the juvenile development for longer from the parent's perspective. The initial broods were set up with 24‐hr‐old larvae, and the larvae were observed as they were 25‐hr‐old. After the observation, the brood was always replaced by a brood of 1‐hr‐old larvae, which were then observed 24 hr later when they were 25‐hr‐old. (c) In the mid‐aged treatment, the larvae remained at the mid‐stage of the juvenile period throughout the experiment. The initial broods were created using 48‐hr‐old larvae, observed at the age of 49 hr, and afterwards always replaced by 25‐hr‐old larvae, which were observed at the age of 49 hr. (d) In the old treatment, the larvae were close to nutritional independence from the beginning to the end of the experiment. The initial broods were created with 72‐hr‐old larvae, observed as they were 73‐hr‐old, and after that always replaced with 49‐hr‐old larvae, which were observed 24 hr later at the age of 73 hr.

We conducted behavioural observations to measure (a) food provisioning provided by the parent to the larvae, (b) begging exhibited by the larvae, and (c) to calculate a compound measure of total amount of time spent on caring behaviours by the parent. The behavioural observations consisted of 30 min of instantaneous scan sampling every 1 min (for details of the protocol, see Smiseth & Moore, 2002). At each scan, we counted the number of larvae begging using the total number of counts across all 30 scans as a measure for begging. At each scan, we also counted the number of larvae in mouth‐to‐mouth contact with the female using the total number of counts across all 30 scans as a measure for provisioning. We also noted whether the female was within a pronotum length's distance from the larvae, as larval begging is triggered only at close proximity to the female (Rauter & Moore, 1999; Smiseth & Moore, 2002). We categorized the behaviour of the female at each scan into seven distinct categories: feeding the larvae, interacting with the larvae, guarding the larvae, maintaining the carcass, consuming the carcass, nonparental behaviours, and being away from the carcass altogether (see Smiseth & Moore, 2002 for definitions). The first four behaviours can be considered as forms of parental care, and as such, we used the total number of counts of all these behaviours across all 30 scans as a compound measure of total care provided by the female. This measure corresponds to the amount of time that a parent spends on caring for the offspring in total, thus being an indication of investment in relation to the time budget of the parent. Due to mortality in the donor broods, experimental females were occasionally discarded in the middle of the experiment, as there were no larvae to provide them with. The behaviour data from any observations before the female was discarded was still used, leading us to have different sample sizes across all time points in the different treatments. Our final sample sizes for each observations conducted at 1, 25, 49 and 73 hr after the larvae were given to the female, were *n* = 21, 21, 21 and 20 for the control treatment; *n* = 39, 36, 35 and 25 for the young treatment; *n* = 26, 25, 20 and 19 for the mid‐aged treatment; and finally *n* = 20, 19, 18 and 18 for the old treatment.

Based on the behavioural data, we measured food provisioning by the parent, larval begging, the total amount of care provided by the parent, and parental responsiveness to begging. The full count of provisioning events within a behaviour observation was used to explore the patterns of resource allocation in the matched and mismatched broods. This parental trait measures the total amount of provisioning for the larvae during the observation period. For investigations of larval begging, we excluded the data from observations where the parent was never close enough to the larvae to trigger begging. A value of 0 for begging events within an observation therefore only relates to observation sessions where larvae did not beg despite there being an opportunity to do so (because the female spent some time in close proximity to them). We thus disregarded the observation sessions where begging did not occur solely because the parent was not there. For this larval trait, we counted the total number of begging events within each observation, and it was used to explore whether larval behaviour was based on cues from the parents or their own age. We also explored parental responsiveness to begging, using data on parental food provisioning and larval begging. To this end, we added larval begging as a fixed effect in our model of female provisioning, thus giving estimates of provisioning relating specifically to the provisioning in response to the amount of begging towards the parent. As before, for this behaviour, we excluded observation sessions where no provisioning happened because the parent was never in close proximity to the larvae. The total sample sizes for the subset of the data that was used for begging and reponsiveness to begging in the different treatments were *n* = 14, 17, 15, 8 for the control treatment; *n* = 23, 23, 21, 7 for the young treatment; *n* = 21, 8, 14, 11 for the mid‐aged treatment; and *n* = 17, 12, 17, 15. for the old treatment, for the observations at 1, 25, 49, and 73 hr after the larvae were given to the females, respectively.

### 2.3 Statistical analyses

All analyses were conducted with R version 3.3.3 (R Core Team, 2017). We used generalized linear mixed effects models for traits with negative binomial error distribution (begging, package lme4; Bates, Maechler, Bolker, & Walker, 2014), and zero‐inflated negative binomial error distributions (provisioning, responsiveness, care, package glmmADMB; Fournier et al., 2012; Skaug, Fournier, Bolker, Magnuson, & Nielsen, 2014), and general linear mixed effect models for traits with Gaussian error distributions (dispersal age, package lme4; Bates et al., 2014). Only up to 24 broods could be taken through the experiment at any given time, and thus the experiment was repeated in six time blocks. For all analyses, we assigned block (to account for non‐independence) and the identity of the female (to account for pseudoreplication) as random factors.

#### 2.3.1 Changes in behaviours over the experimental period

In models exploring changes in the levels of behaviour traits (provisioning, begging, total care) over the experimental period, we assigned experimental treatment (control, young, mid‐aged or old), time of observation (1, 25, 49 or 73 hr after the first brood was given to the female), and the interaction between the two, as fixed effects. For all the behaviours we measured, our main goal was to compare the patterns of behaviours over time between the control treatment and the experimental treatments. To this end, we used the interaction terms between treatment and observation time to observe these changes: At a given interaction level *Treatment*(*x*)*:Time*(*y*), the statistics provided below show how the change from Time(0) to Time(*y*) differs between the control treatment and Treatment(*x*). Thus, significant differences infer that the direction or magnitude of the change over time in the level of a certain behaviour in Treatment(*x*) deviates from that of the control treatment, and therefore that the pattern of the behaviour is different.

#### 2.3.2 Parental responsiveness to begging

To explore parental responsiveness to begging, we generated a model with provisioning events as a response variable, and begging events as a covariate, using only the data from observations where the parent was close enough to the larvae to experience begging (see Experimental design). As we were interested in how responsiveness changes in the different treatments and observation times, we also added these two factors in as fixed factors, as well as their respective interactions with begging behaviour.

#### 2.3.3 Dispersal time

For exploring the age of the larvae at dispersal, we assigned treatment and the total amount of care given during the last observation, as well as the interaction between the two as fixed effects. We used the care given at the last observation as a proxy for the amount of care received by the offspring, as the larvae of this observation were the same ones that were dispersing. Female ID was dropped from the random effects structure in this model, as there were no repeated measures for brood age at dispersal.

## 3 RESULTS

### 3.1 Changes in behaviours over the experimental period

#### 3.1.1 Provisioning

**FIGURE 2:**
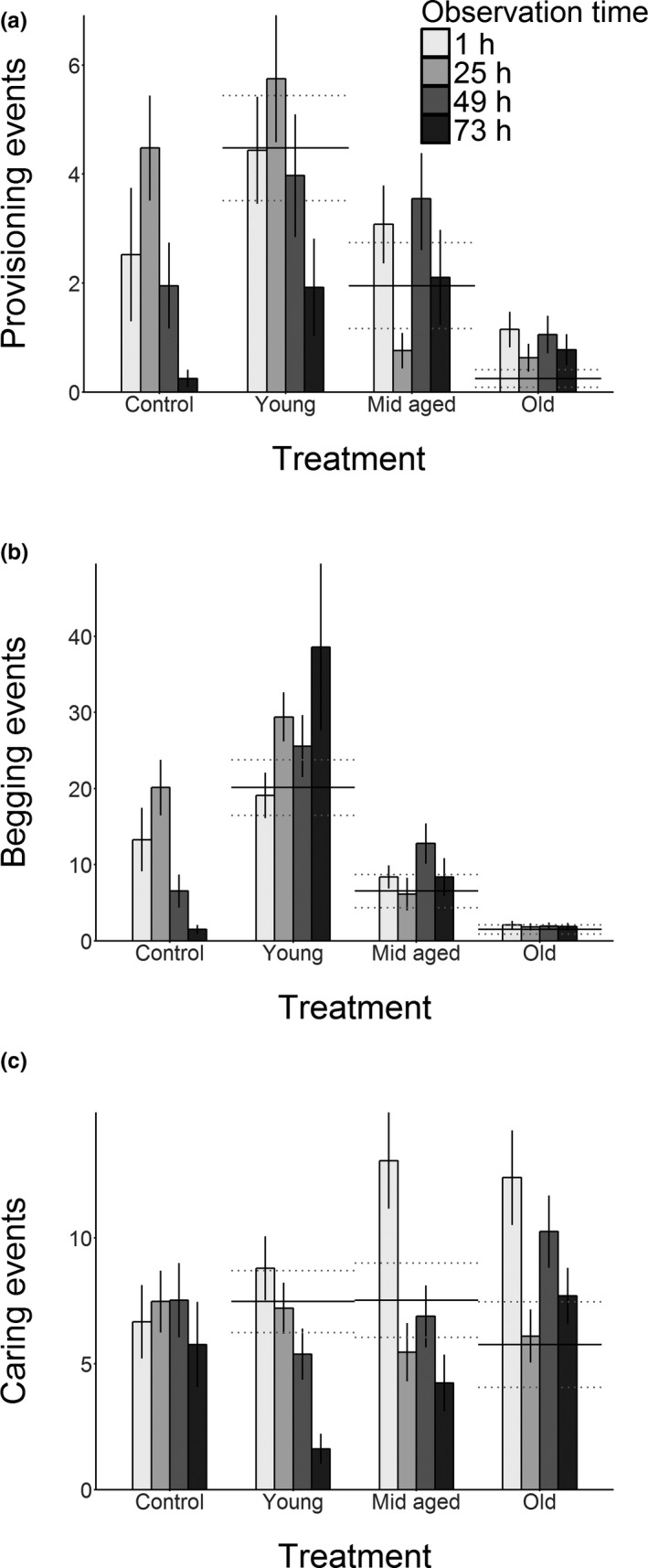
Mean (±*SE*) of behaviour traits related to resource allocation, observed during 30‐min behaviour observation conducted in 24‐hr interval. (a) Count of provisioning events during the observation. (b) Count of the number of larvae begging during the observation. (c) Count of caring events during the observation. Dark line over each experimental treatment corresponds to the behaviour levels of the treatment with same‐aged larvae in the control treatment and its *SE* (dotted lines)

In the control treatment, the provisioning behaviour changed over time in the following pattern: the behaviour peaked during the second observation (25 hr after the perceived hatching), and declined thereafter showing very low levels during the last observation (73 hr after the perceived hatching; Figure 2a). This is a pattern comparable with previous findings on the same species (Smiseth et al., 2003). Overall, the pattern of parental food provisioning in the control treatment was different from the three experimental treatments (interaction term in Table 1, Figure 2a). In the young treatment, the pattern of total provisioning resembled that of the control, but with the notable difference that the larvae were provisioned more at the end of the experiment (see interaction term Young:Time(73) in Table 1, Figure 2a). The higher levels of provisioning at this stage suggest that females adjust their behaviour to the age of the larvae, possibly to accommodate the higher needs of young larvae. In the mid‐aged treatment, provisioning to the larvae fluctuated over time (Table 1, Figure 2a), but the levels of provisioning remained on average at the same level throughout the experiment (Figure 2a). In the old treatment, the levels of provisioning also remained stable throughout the experiment, with the larvae being fed more than the larvae at the same stage in control treatment overall (Table 1, Figure 2a).

**TABLE 1:** Changes in behaviour traits of the burying beetle *Nicrophorus vespilloides* over the duration of the period of parental care in treatments where the age the parents expected their brood to be, and the actual age of the brood were experimentally mismatched

Factor	Provisioning			Begging			Total care		
	Par (*SE*)	*t*/χ^2^	*p*	Par (*SE*)	*t*/χ^2^	*p*	Par (*SE*)	*t*/χ^2^	*p*
Treatment		18.46	<.001		178.90	<.001		31.60	<.001
Young	0.19 (0.39)	0.48	.635	0.43 (0.31)	1.41	.157	0.38 (0.23)	1.61	.107
Mid‐aged	−0.52 (0.40)	−1.29	.197	−0.41 (0.31)	−1.30	.194	0.76 (0.25)	3.07	.002
Old	−1.49 (0.46)	−3.25	.001	−1.78 (0.36)	−4.95	<.001	0.68 (0.26)	2.59	.009
Observation time		14.53	.002		7.01	.071		0.80	.850
Time(25)	−0.10 (0.39)	−0.26	.796	0.49 (0.31)	1.56	.119	0.00 (0.22)	0.00	.998
Time(49)	−0.69 (0.44)	−1.58	.114	−0.71 (0.33)	−2.17	.030	0.15 (0.23)	0.66	.508
Time(73)	−2.66 (0.65)	−4.07	<.001	−2.09 (0.48)	−4.40	<.001	0.22 (0.25)	0.87	.387
Treatment:Observation time		45.70	<.001		39.18	<.001		71.93	<.001
Young:Time(25)	0.24 (0.46)	0.52	.600	−0.06 (0.40)	−0.14	.886	−0.17 (0.27)	−0.60	.546
Mid‐aged:Time(25)	−0.82 (0.59)	−1.40	.161	−0.82 (0.49)	−1.67	.095	−0.80 (0.30)	−2.69	.007
Old:Time(25)	−0.18 (0.64)	−0.29	.774	−0.59 (0.51)	−1.14	.254	−0.69 (0.31)	−2.20	.028
Young:Time(49)	0.54 (0.51)	1.05	.292	1.00 (0.42)	2.40	.016	−0.58 (0.29)	−1.97	.049
Mid‐aged:Time(49)	0.84 (0.53)	1.59	.111	1.09 (0.44)	2.45	.014	−0.74 (0.30)	−2.45	.014
Old:Time(49)	0.81 (0.62)	1.30	.192	0.63 (0.49)	1.28	.200	−0.39 (0.31)	−1.28	.201
Young:Time(73)	2.66 (0.76)	3.48	<.001	2.76 (0.60)	4.60	<.001	−1.20 (0.45)	−2.69	.007
Mid‐aged:Time(73)	2.62 (0.75)	3.50	<.001	2.05 (0.58)	3.52	<.001	−1.05 (0.35)	−2.98	.003
Old:Time(73)	2.48 (0.81)	3.05	.002	1.96 (0.61)	3.21	.001	−0.69 (0.33)	−2.09	.036

Note

Estimates are derived from generalized linear mixed effects models with experimental block and the identity of the female assigned as random factors. We present parameter estimates (and *SE*), *t*‐statistics and *p*‐values for each factor level, as well as χ^2^ statistics and *p*‐values for the overall effects of each factor. The degrees of freedom were estimated with Satterthwaite approximation.

#### 3.1.2 Begging

The control treatment followed the same pattern as the female's provisioning behaviour (see above), and as described in previous studies on this species (Smiseth et al., 2003, Figure 2b). The pattern of begging suggests that there is little plasticity in larval behaviours, as the levels of begging remained constant at a level determined by the age of the larvae only (Figure 1b). In the young treatment, however, the amount of begging increased throughout the experimental period (Table 1, Figure 2b). The pattern of begging in the mid‐aged treatment also differed from the pattern of the control treatment (Table 1), with begging fluctuating around the level of begging exhibited by the same‐aged larvae of the control treatment (Figure 1b). Finally, the old larvae begged at low levels overall throughout the experiment, and the level of begging was approximately constant (Figure 2b).

#### 3.1.3 Total care

In the control treatment, females provided the same amount of overall care at all stages of juvenile development (Figure 2c). However, the patterns of total amount of care given in each experimental treatment deviated from that of the control treatment (interaction term in Table 1, Figure 2c). When larval begging declined as the larvae grew older, females tended to switch to indirect forms of care (i.e. maintaining the carcass or guarding the larvae), as evident by the decline in provisioning behaviour (Figure 2a), but maintained stable levels of total care (Figure 2c, see also Appendix S1). In the young treatment, the total amount of care declined over time (Table 1, Figure 2c). Both the mid‐aged and old treatments showed a peak in care behaviours during the first observation (Table 1, Figure 2c). This was in part due to the females interacting with the larvae directly in ways other than provisioning (such as grooming the larvae) more often than in the control treatment during these observations. While grooming behaviour is rare in general, we observed the parents grooming the larvae on 46 occasions in the mid‐aged, and 95 occasions in the old treatment, when similar observations were only made 10 times in the control, and three times in the young treatment (Appendix S1).

### 3.2 Parental responsiveness to begging

**FIGURE 3:**
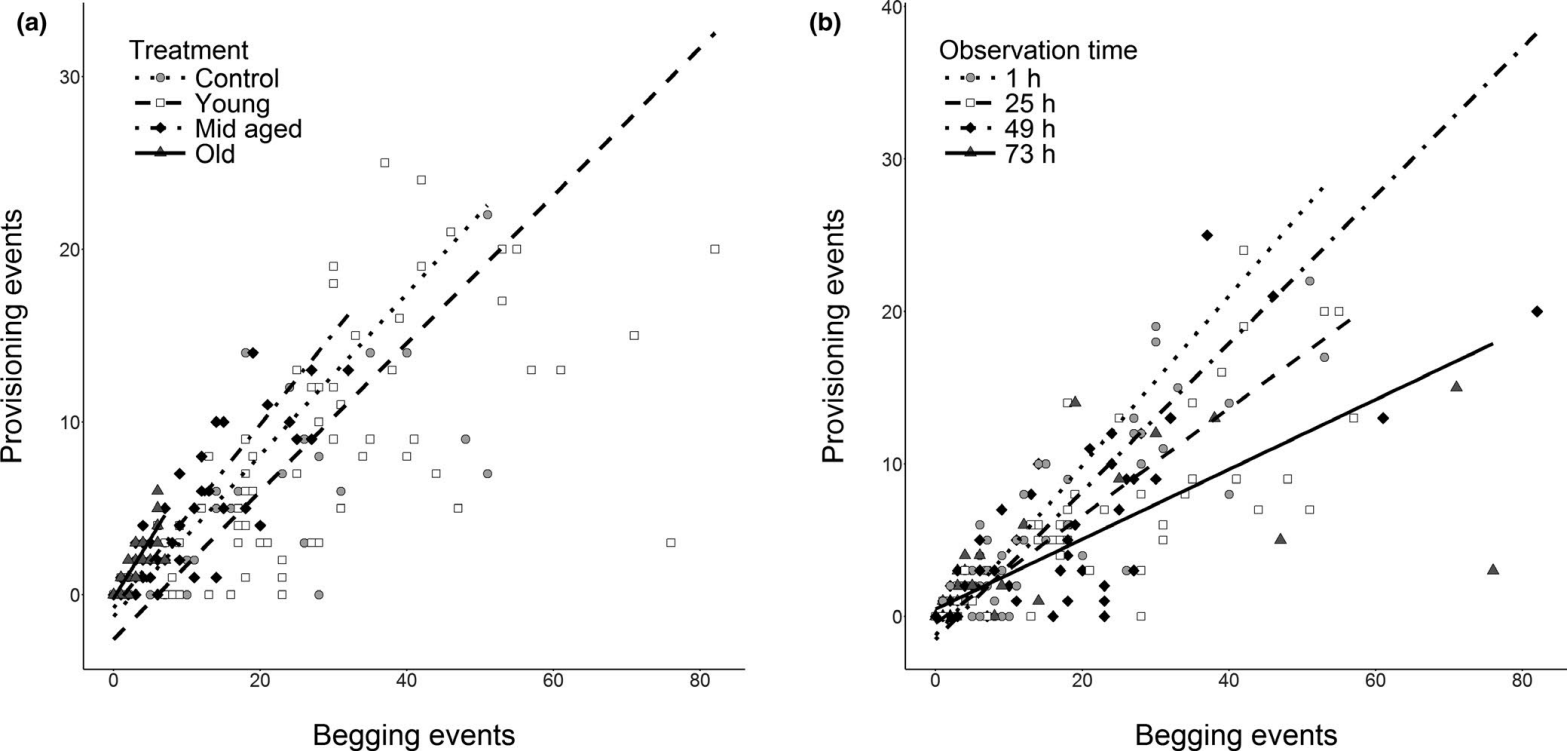
Changes in parental responsiveness to begging in (a) different treatments, and (b) observation times. Steeper slopes represent higher responsiveness. The points represent raw data, and the regression lines are derived from the fitted values of generalized linear mixed effects models. Only data from observations where the parent was in the presence of the larvae were included in this analysis

Overall, there was a positive association between the amount of larval begging and female food provisioning in all treatments (Figure 3a) and observation times (Figure 3b). In the young treatment, parental responsiveness to begging remained similar to the control treatment (Treatment:Begging interaction in Table 2, Figure 3a). In the mid‐aged and old treatments the responsiveness was higher than in the control treatment, as an increase in begging increased parental food provisioning to a bigger extent than it did in the control treatment (Treatment:Begging interaction in Table 2, Figure 3a). However, the levels of provisioning and begging were relatively low in the old treatment and the increase was very small in the mid‐aged treatment, indicating that even with the increased responsiveness, the provisioning levels remained much lower than they were in the control treatment (Figure 3a). The responsiveness declined overall from the first observation to the last (Observation time:Begging interaction in Table 2, Figure 3b).

**TABLE 2:** Parental provisioning as a response to experienced begging in the burying beetle *Nicrophorus vespilloides* in treatments where the parental expectations and offspring need were mismatched through manipulations of brood age

Factor	Par (*SE*)	*t*/χ^2^	*p*
Begging		36.51	<.001
Begging	0.08 (0.01)	7.16	<.001
Treatment		6.03	.110
Young	0.58 (0.27)	2.13	.033
Mid‐aged	0.15 (0.29)	0.53	.597
Old	−1.31 (0.35)	−3.72	<.001
Observation time		1.18	.758
Time(25)	0.34 (0.24)	1.43	.153
Time(49)	0.12 (0.23)	0.51	.609
Time(73)	0.01 (0.25)	0.03	.973
Treatment:Begging		35.24	<.001
Young:Begging	−0.02 (0.01)	−1.56	.118
Mid‐aged:Begging	0.04 (0.02)	2.10	.036
Old:Begging	0.42 (0.08)	5.33	<.001
Observation time:Begging		14.29	.003
Time(25):Begging	−0.02 (0.01)	−1.99	.046
Time(49):Begging	−0.02 (0.01)	−1.80	.073
Time(73):Begging	−0.03 (0.01)	−2.59	.010

Note

Estimates are derived from a generalized linear mixed effects model with experimental block and the identity of the female assigned as random factors. We present parameter estimates (and *SE*), *t*‐statistics, and *p*‐values for each factor level, as well as χ^2^ statistics and *p*‐values for the overall effects of each factor. The degrees of freedom were estimated with Satterthwaite approximation.

### 3.3 Dispersal time

Overall, the ages at which larvae dispersed from the carcass did not differ between treatments (lmer, *F*_3,67_ = 2.17, *p* = .099). However, the larvae of the young treatment did disperse from the carcass at a slightly younger age than the control offspring (Parameter estimate (±*SE*): −0.34 (±0.17), *p* = .049). No statistical differences in dispersal age were found between the control treatment and the mid‐aged (Parameter estimate (±*SE*): 0.06 (±0.20), *p* = .784) or the old treatment (Parameter estimate (±*SE*): 0.07 (±0.28), *p* = .802). Therefore, while this indicates that larvae in the young treatment dispersed on average earlier than the control larvae, the actual detected differences were so small, that they are unlikely to be biologically meaningful. There was no overall relationship between the amount of care given and the age at dispersal (lmer, *F*_1,68_ = 0.67, *p* = .417), and these relationships did not differ between treatments (lmer, *F*_3,67_ = 0.84, *p* = .476). We note that, due to our experimental design, larvae in different treatments did not experience the same conditions throughout their development. We therefore urge caution when interpreting our results for larval development time.

## 4 DISCUSSION

In exploring the contrasting assumptions made by theoretical models on the resolution of parent–offspring conflict (Godfray, 1991; Parker et al., 2002), we found that control over care shifted from offspring to parents over the course of the period of dependency. Neither parental food provisioning nor offspring begging followed either of the predicted hypothetical extremes of full parental or full larval control (Figure 1). Parents showed more plasticity in response to cues from offspring age, while the larvae largely begged according to their own age (Figure 2a,b), indicating some support for larval control over resource allocation. However, due to declines in parental responsiveness to begging (Figure 3) and in the amount of care given to the young (and thus neediest) broods (Figure 2a,c), larval control seems to decline when the broods approached transitioning to nutritional independence. Thus, the major patterns in these behaviours indicate support for mutual control over resource allocation. Below we discuss our results from the perspectives of both models in more detail. We also consider the role of plasticity in conflict resolution from an evolutionary point of view.

According to scramble competition models, resource allocation within the brood is determined by sibling competition, and the parent responds to signals received from the offspring (Parker et al., 2002). In this experiment, this assumption would have been supported by full correspondence between parental food provisioning and offspring begging. Our data partially support this model, as the parents behaved largely plastically, and especially during the first observations, adjusted their provisioning (Figure 2a) to the levels of begging exhibited by the larvae (Figure 2b) across all treatments, indicating offspring control over resource allocation during these observations. Besides a strong response to begging cues, we found that the total amount of care given by females was higher when caring for mid‐aged and old larvae, and the amount of care declined over time when caring for young larvae. This pattern may be explained by the perceived differences in the quality of the offspring and their value to the parent. For example, in the European earwig (*Forficula auricularia*), mothers who had been exposed to chemical cues of either broods with high food availability or low food availability, provided more care when they perceived their brood to be of high quality, and showed more aggression towards the offspring when they perceived their brood to be of low quality (Mas & Kölliker, 2011). Evidence from studies on *N. vespilloides* also suggests that parents respond to cues other than begging when determining the amount of provisioning, as the parents provision more food to inbred offspring, although the same offspring also beg less than their outbred counterparts (Mattey, Richardson, Ratz, & Smiseth, 2018). Similarly, adult *N. vespilloides* are known to adjust their behaviour to the number of larvae in a brood (Ratz & Smiseth, 2018; Smiseth & Moore, 2007). In our study, the older larvae of the mid‐aged and old treatments may have been assessed as being of good quality, potentially due to their size or associated traits, thereby prompting the female into elevating the amount of care beyond the level provided by control females. This finding suggests that the females respond to cues of offspring need and quality (aside from begging) and that parents plastically adjust the total amount of time spent caring when the expected age of the brood from the female's perspective is mismatched with the actual age of the larvae. However, whether females respond plastically to their own benefit or that of the offspring, is an open question.

In honest signalling models (Godfray, 1991), parents control resource allocation: The signals from the offspring, whether behavioural or visual, are expected to be an honest indication of the nutritional state of the offspring, thus allowing the parent to allocate resources where they are most needed, ensuring maximum returns on their investment in the offspring. Signalling is costly, and the benefits from it vary with offspring need (Godfray, 1991). In the burying beetle *N. vespilloides*, previous work has shown that begging conveys information about the hunger state of the larvae (Smiseth & Moore, 2004), thus indicating it is an honest signal of need. Additionally, begging is costly through increased risk of parental cannibalism (Andrews & Smiseth, 2013). Our data indicate that offspring beg according to their age (and thus, state of need; Figure 2b, Table 1), also supporting the honesty of begging signals. Further indication of active resource allocation choices from the parents is that the parents provision more to the larvae in the old treatment, despite no statistical differences in the amount of begging they exhibit in comparison to the broods of the same age in the control treatment. Similarly, parents reduce their care (and their food provisioning) over time in the young treatment despite no change in signals received from the offspring. This could be due to a change in the perceived honesty of the signal: According to the honest signalling models, the parent optimizes its fitness by responding to begging when it is a true indication of offspring need (Godfray, 1991, 1995a). Integrating information on the level of begging and the expected age of the brood to infer a certain level of need could ensure that the parent will not be manipulated to provision excessive amount of care through exaggerated begging. Thus, as the parents eventually reduced the amount of care and their responsiveness, and because they overall gave more care to older broods despite no difference in their begging behaviour, provisioning of food seems determined by the parent, lending some support for the honest signalling models.

As discussed above, our results provide evidence for mutual control over resource allocation. We find evidence for offspring control as offspring begged according their own age, while we also find evidence for parental control as parents adjusted their provisioning also to their own benefit. Our results provide some indications of how the balance of power between parents and offspring shifts over time. Both provisioning and begging do closely follow the predictions of offspring control. Meanwhile, parental responsiveness to begging had a shallower slope for later observation time points, which is consistent with evidence for a shift towards more parental control later in the dependent period. Nevertheless, we note that females spent more time provisioning food at the final observation in all three treatment groups than in the control group. This suggests that offspring can still extract more provisioning from the parents than they normally would optimally provide at this time point. At the 1‐hr time point, for example, control parents (receiving the coadapted rate of begging) perform an average of 2.5 provisioning events, which increases 1.8‐fold to 4.5 provisioning events for the same time point in the young treatment (where the begging rate has increased 1.4‐fold). The same comparison for the 73‐hr time point finds that the provisioning rate increases 9.5‐fold, from 0.2 to 1.9 provisioning events (with a 25.5‐fold increase in begging). While the provisioning per begging effort is certainly less (as shown by Figure 3b), the increase in provisioning from the coadapted baseline is far greater, and can thus be seen as support for larval control. This is not to say that parents have no control, however, as comparing these levels of provisioning to the levels of provisioning based on actual larval age (lines across each experimental treatment in Figure 2a) shows that the parents do not fully satiate offspring needs either. The lack of correspondence between observed behaviours and the two reference points (i.e. the control treatments at the time of observation and the time corresponding to the age of the larvae), indicates that resources allocation is likely affected by both females and offspring.

An alternative explanation for our observed patterns for food provisioning is that these are driven by energy constraints on females. For example, females assigned to the young treatment provided care towards young larvae that were more dependent on care and begged more over 4 days, while females assigned to the old treatment would have provided care towards old larvae that were less dependent and begged less over the same time period. Thus, the former females would have been working closer to their maximum capacity over a sustained period, and if care is costly, this may have caused them to reduce their care towards the end of the experiment. However, we argue that this explanation is unlikely in our study species. Firstly, there is good evidence that females have at least some capacity to increase their level of care. For example, prior work shows that females increase their level of care in response to brood size enlargement and that they do over the duration of our experiment (Smiseth & Moore, 2007). We also note that we used a relatively small brood size (10 larvae) in our experiment, which suggests that females in all treatments would have had the capacity to increase their level of care in response to a change in the actual age of the larvae. Secondly, there is little evidence that care incurs detectable energetic costs to females in our study species (Richardson, Stephens, & Smiseth, 2020). This is likely to reflect that females provision their larvae with food from a resource that has been acquired prior to breeding (i.e. the carcass of a small vertebrate), and that females also feed from carcass and thereby replenish their body reserves during breeding (Richardson et al., 2020). However, we note that such constraints may play an important role in species where parents make repeated foraging trips to the brood, such as in birds. Thus, it is important to acknowledge the potential role of time and energy constraints on females when interpreting results from the design used in our study.

For the parent, responses to signals received from the offspring may be dependent on the fluctuations in the costs and benefits of care, leading to a continuum where the control over resource allocation shifts from the offspring at the time of hatching to the parent at the time of nutritional independence (Royle et al., 2002). The decline in care and responsiveness observed in our data for the youngest offspring could be due to the costs of care exceeding the benefits that investment into the current broods has the potential to provide. Similarly, female burrower bugs (*Sehirus cinctus*) become less responsive if their eggs hatch too early, but remain attentive for longer than in normal setting when the eggs take too long to hatch, reflecting the changes in the pay‐offs of caring for the offspring or producing a new brood (Kight, 1997). Furthermore, while there is evidence that parents alter their provisioning behaviour in response to cues from the offspring in a wide range of taxa (Bell, 2008; Bruce, 1958; Rehling et al., 2012), the parents often reduce their response after a given time (Lucass, Stowe, Eens, & Muller, 2016; Rehling & Trillmich, 2007; Riou et al., 2012), presumably to limit the future costs of care. Our data could imply a similar strategy employed by the female burying beetles, where responsiveness declines to avoid costs to future reproduction. Mechanistically, this adjustment may be hormonally controlled, as the levels of juvenile hormone stay at a high level when females (Scott & Panaitof, 2004) or males (Trumbo & Robinson, 2008) of the burying beetle (*Nicrophorus orbicollis*) encounter young offspring for longer than would occur in a natural setting, but eventually return to normal. The parent thus needs to balance the benefits and costs of plasticity in terms of its effects on their own survival and reproduction against its effects on its offspring's survival and reproduction.

Considering that it takes a prolonged disruption in the rates of begging, caused by repeated mismatching in our experiment, for the parents to alter their behaviour towards the offspring and that the treatments did not alter the age at which the larvae become fully independent of their parent, one possible explanation is that there is little conflict battleground left in *N. vespilloides* and that both parent and offspring are already close to their optima. Since the introduction of honest signalling models (Godfray, 1991), the presence of offspring begging has often been taken as a signature of parent–offspring conflict (i.e. divergence in the two optima), as begging is assumed to be an evolutionary stable behavioural mechanism for conflict resolution. However, there is little evidence to show that there is divergence in the two optima. If the two optima are similar, offspring begging may simply serve the role of coordinating parental behaviours with the offspring's needs. From this point of view, it is important to question whether this resolution and the age‐dependent coadaptation serve the evolutionary interests of parents, offspring or both. As our results suggest, the realized control over resource allocation may often lie between the two extremes of full control, with the power shifting from parents to offspring throughout offspring development (Royle et al., 2002). The majority of the literature has previously indicated that parents have the upper hand in determining the amount of resources given (Hinde et al., 2010; Kölliker, Brodie, & Moore, 2005; Lucass et al., 2016; Thorogood, Ewen, & Kilner, 2011; Wong, Lucas, & Kölliker, 2014). However, as the amount of parental care given changes both based on the offspring's needs (this study, Djerdali et al., 2008; Price, 1998; Rehling et al., 2012; Wong et al., 2014), and of the parents' own state (Thorogood et al., 2011; Wong & Kölliker, 2012), parental care is evidently plastic to cues from both parties. Mutual plasticity where parents and offspring adjust their behaviours to each other, and constraints on plasticity, such as females responding differently to offspring begging depending on the offspring's age, may leave individuals less vulnerable to exploitation by the other party. Thus, this type of plasticity can function to alleviate conflict rather than enable it. Plasticity is thus likely to allow both the parent and the offspring to get closer to their own optimal level of investment, in a compromise solution that is mutually beneficial to both parties.

## AUTHOR CONTRIBUTIONS

M.I. M. and P.T.S. conceived the idea and designed the experiment. M.I. M. carried out the laboratory work, analysed the data and lead the writing of the manuscript. Both authors contributed to the revisions.

### Peer Review

The peer review history for this article is available at https://publons.com/publon/10.1111/jeb.13692.

## Supporting information

Appendix S1

## Data Availability

Data available on Dryad at https://doi.org/10.5061/dryad.z08kprr9q
