# Supplementary material for: Resource allocation is determined by both parents and offspring in a burying beetle
Source: J Evol Biol. 2020 Sep 14;33(11):1567–78. doi: 10.1111/jeb.13692 (PMC7692937; doi:10.1111/jeb.13692)
Supplement: Supplementary file 1 — Appendix S1 [file JEB-33-1567-s001.pdf]

Supplementary material S1: Analyses of other parental behaviours

In each behaviour observation conducted during the experiment, we categorised the behaviour of the female *Nicrophorus vespilloides* parent at each scan into seven distinct possibilities: feeding the larvae, interacting with the larvae, guarding the larvae, maintaining the carcass, consuming the carcass, nonparental behaviours, and being away from the carcass altogether. We also recorded whether the female was within a pronotum's length distance away from the larvae, thus being close enough to them to enable begging (trait hereafter referred to as proximity). Each behaviour was analysed separately to investigate any potential confounding factors in the data. We do not present the data for feeding separately, as it is very close to the data for provisioning: For feeding we only counted the number of scans when the female was provisioning, whereas counts for provisioning also take into account the number of larvae being provisioned for at any given time. Here we present results of analyses on behaviours that were not presented in the main body of the article: interacting with the larvae, indirect care (see below for the definition), consuming the carcass, non-parental behaviours and time spent away, as well as female's proximity to the larvae.

We summarised behaviours that consisted of the female providing care to the offspring indirectly (guarding the larvae and maintaining the carcass) into one category. This was done as guarding is a very rare behaviour, and it was observed equally rarely in all observations conducted and provided no additional insight into the behaviours exhibited by the females. Non-parental behaviours encompassed a multitude of behaviours such as the female grooming itself, ruminating, walking or hiding under the carcass, all of which were categorized as non-parental during the observations. All traits were treated as counts.

Statistical analyses

All analyses were conducted with R version 4.0.0 [1]. The behaviour traits had zero-inflated (interacting with the larvae, consuming the carcass, proximity) and non-zero-inflated (indirect care, non-parental behaviours, time spent away) negative binomial error structures, and as such they were analysed using generalized linear mixed-effects models (R package glmmADMB, [2,3]). In all models, we assigned experimental treatment (control, young, mid-aged or old), time of observation (1 hour, 25 hours, 49 hours or 73 hours after the start of the experiment), and the interaction between the two, as fixed effects, and block and the identity of the female (to control for pseudoreplication) as random factors.

Results

Interacting with the larvae

Interactions with the larvae varied both based on the baseline of the treatment, and the time of the observation (Table S1.1). The largest deviation from the pattern observed in the control treatment was the very high levels of interactions observed during the first observation in the mid-aged and old treatments (Table S1.1, Fig. S1.1).

Indirect care

Overall, the pattern of indirect care was different in the control treatment than in the old experimental treatment (Table S1.2). In the young treatment, the amount of indirect care provided declined towards the end of the experiment (similarly to the amount of total care presented in the main body of the article; Table S1.2, Fig. S1.2). Amount of indirect care provided was more stable in the mid-age treatment, although it was still declining, and the amount of indirect care provided fluctuated in the old treatment (Table S1.2, Fig. S1.2). The changes in indirect care behaviours are primarily due to changes in the amount of time the female spent maintaining the carcass, and the general pattern corresponds to that found for total care.

Consuming the carcass

All treatments aside from the mid-aged one followed the pattern of the control treatment for the time spent on consuming the carcass (Table S1.3). The pattern of the mid-aged treatment was more unstable, and the females consumed more of the carcass at the end of the experiment than the females in the control treatment (Table S1.3, Fig. S1.3). Aside from the peak in mid-aged treatment, no deviations from the pattern of the control treatment were detected in the frequency of consuming the carcass.

Non-parental behaviours

The pattern of non-parental behaviours was different in some of the experimental treatments than it was in the control treatment (Table S1.4). The females in the control treatment spent less time on non-parental behaviours during the observation conducted 1 h and 73 h into the experiment (Table S1.4, Fig. S1.4). The young treatment followed the pattern of the control treatment, but a different pattern was observed for mid-age and old treatments (Table S1.4, Fig. S1.4). Both mid-age and old treatments started at lower levels of non-parental behaviours, but the time spent on these behaviours increased in the subsequent observations (Table S1.4, Fig. S1.4). Females in the mid-aged and old treatments also spent less time on non-parental behaviours in total (Table S1.4), which was to be expected based on the the same females spending more time on total care (see main text of the article).

Time spent away

Females of the young treatment spent more time away overall (Table S1.5)., and all experimental females spent more time away from the carcass at 25 h after the start of the experiment (Fig. S1.5). However, no statistically significant deviation from the changes in the levels of behaviours of the control treatment was detected in the experimental treatments (Table S1.5).

Time spent in the proximity of the larvae

There were no distinct differences in the time spent in the proximity of the larvae between the control treatment and the young and old treatments (Table S1.6, Fig. S1.6). The females of the mid-aged treatment spent less time in the proximity of the larvae in the observation conducted at 25 h after the start of the experiment, but aside from that there were no differences between the mid-aged treatment and the control treatment (Table S1.6, Fig. S1.6).

Concluding remarks

All behaviour traits presented here were accounted for in the count for total care provided presented in the main text of the article. Indirect care was a part of total care provided, encompassing all behaviours that were not accounted for by the time spent interacting with the larvae (presented in the paper) and feeding. The rest of the behaviours presented here - consuming the carcass, non-parental behaviours, and time spent away - were accounted for as the opposite of total care. These three behaviours and total care were mutually exclusive. There were no contradictory patterns in the comparisons between the control treatment and the experimental treatments in these three behaviours, suggesting that our measure of total care provided encompassed the main differences in the types of behaviours exhibited by the females. There was also no statistically significant pattern detectable in the time spent within proximity of the larvae, hence showing that the females were equally likely to be close to the larvae in all treatments, and the differences detected in the behaviours were due to the female behaviour rather than their proximity to the larvae.

Table S1.1. Occurences of the female burying beetles *Nicrophorus vespilloides* interacting with the larvae in ways other than feeding during the 30 min behaviour observations. For each factor on the treatment level, there is information on parameter estimate (Par), standard error (SE), test statistic (Z value), and the P value (P). Analyses were conducted with generalized linear mixed effects models (glmmADMB) with experimental block and the identity of the female assigned as random factors.

Factor	Par	SE	Z value	P
Young	0.670	0.567	1.183	0.237
Mid-aged	2.232	0.541	4.125	0.00004
Old	2.604	0.548	4.753	0.00000
Time(25)	1.100	0.592	1.858	0.063
Time(49)	1.078	0.591	1.825	0.068
Time(73)	1.058	0.610	1.734	0.083
Young:Time(25)	-1.304	0.724	-1.801	0.072
Mid-aged:Time(25)	-2.523	0.722	-3.494	0.0005
Old:Time(25)	-2.465	0.713	-3.455	0.001
Young:Time(49)	-1.584	0.743	-2.133	0.033
Mid-aged:Time(49)	-1.956	0.714	-2.742	0.006
Old:Time(49)	-2.754	0.719	-3.829	0.0001
Young:Time(73)	-1.192	0.766	-1.556	0.120
Mid-aged:Time(73)	-2.370	0.746	-3.177	0.001
Old:Time(73)	-1.971	0.703	-2.801	0.005

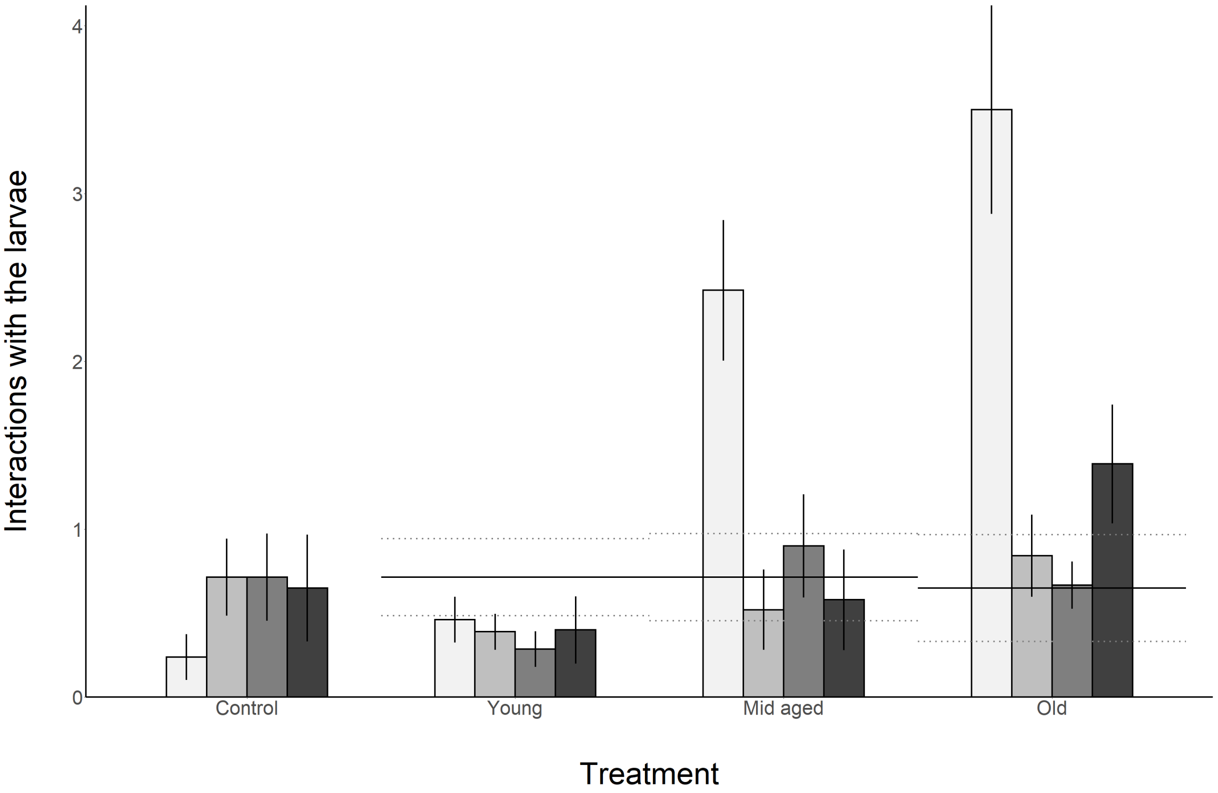

Fig. S1.1. Mean (± standard error) of the counts of occurences the female burying beetles *Nicrophorus vespilloides* interacting with the larvae during the 30 min behaviour observations conducted in 24 hour interval. Dark line over each experimental treatment corresponds to the behaviour levels of the treatment with same-aged larvae in the control treatment and its standard error (dotted lines).

Table S1.2. Indirect care behaviours exhibited by the female burying beetle *Nicrophorus vespilloides* during the 30 min behaviour observations. For each factor on the treatment level, there is information on parameter estimate (Par), standard error (SE), test statistic (Z value), and *P* value (*P*). Analyses were conducted with generalized linear mixed effects models (glmmADMB) with experimental block and the identity of the female assigned as random factors.

Factor	Par	SE	Z value	*P*
Young	0.249	0.306	0.813	0.416
Mid-aged	0.521	0.328	1.589	0.112
Old	0.578	0.347	1.666	0.096
Time(25)	-0.184	0.306	-0.603	0.546
Time(49)	0.195	0.306	0.637	0.524
Time(73)	0.017	0.310	0.056	0.956
Young:Time(25)	-0.100	0.381	-0.262	0.793
Mid-aged:Time(25)	-0.376	0.407	-0.925	0.355
Old:Time(25)	-0.244	0.428	-0.570	0.569
Young:Time(49)	-0.780	0.386	-2.021	0.043
Mid-aged:Time(49)	-0.587	0.418	-1.405	0.160
Old:Time(49)	0.078	0.426	0.184	0.854
Young:Time(73)	-1.947	0.444	-4.384	0.00001
Mid-aged:Time(73)	-0.821	0.433	-1.895	0.058
Old:Time(73)	-0.150	0.431	-0.349	0.727

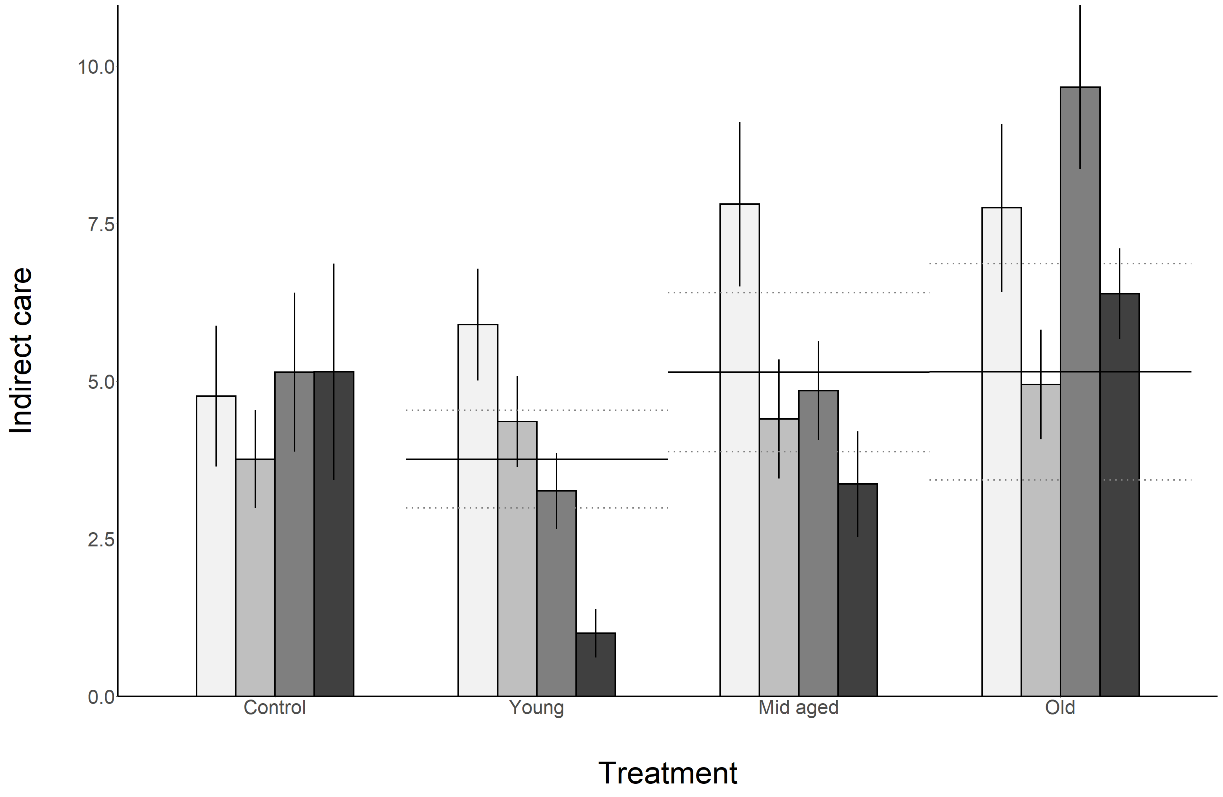

Fig. S1.2. Mean (± standard error) of the counts of occurences the female burying beetles *Nicrophorus vespilloides* caring for their offspring indirectly (maintaining the carcass or guarding the larvae) during the 30 min behaviour observations conducted in 24 hour interval. Dark line over each experimental treatment corresponds to the behaviour levels of the treatment with same-aged larvae in the control treatment and its standard error (dotted lines).

Table S1.3. Occurences of the female burying beetles *Nicrophorus vespilloides* consuming the carrion during the 30 min behaviour observations. For each factor on the treatment level, there is information on parameter estimate (Par), standard error (SE), test statistic (Z value), and the P value (P). Analyses were conducted with generalized linear mixed effects models (glmmADMB) with experimental block and the identity of the female assigned as random factors.

Factor	Par	SE	Z value	P
Young	0.248	0.389	0.639	0.523
Mid-aged	-0.156	0.405	-0.386	0.700
Old	-0.281	0.463	-0.606	0.544
Time(25)	0.357	0.335	1.068	0.285
Time(49	0.112	0.377	0.297	0.767
Time(73	-0.654	0.549	-1.190	0.234
Young:Time(25)	-0.191	0.416	-0.460	0.645
Mid-aged:Time(25)	0.063	0.524	0.121	0.904
Old:Time(25)	0.526	0.488	1.078	0.281
Young:Time(49	-0.134	0.461	-0.290	0.772
Mid-aged:Time(49	0.640	0.465	1.376	0.169
Old:Time(49	0.439	0.535	0.820	0.412
Young:Time(73	0.477	0.698	0.684	0.494
Mid-aged:Time(73	1.419	0.627	2.263	0.024
Old:Time(73	0.409	0.752	0.544	0.586

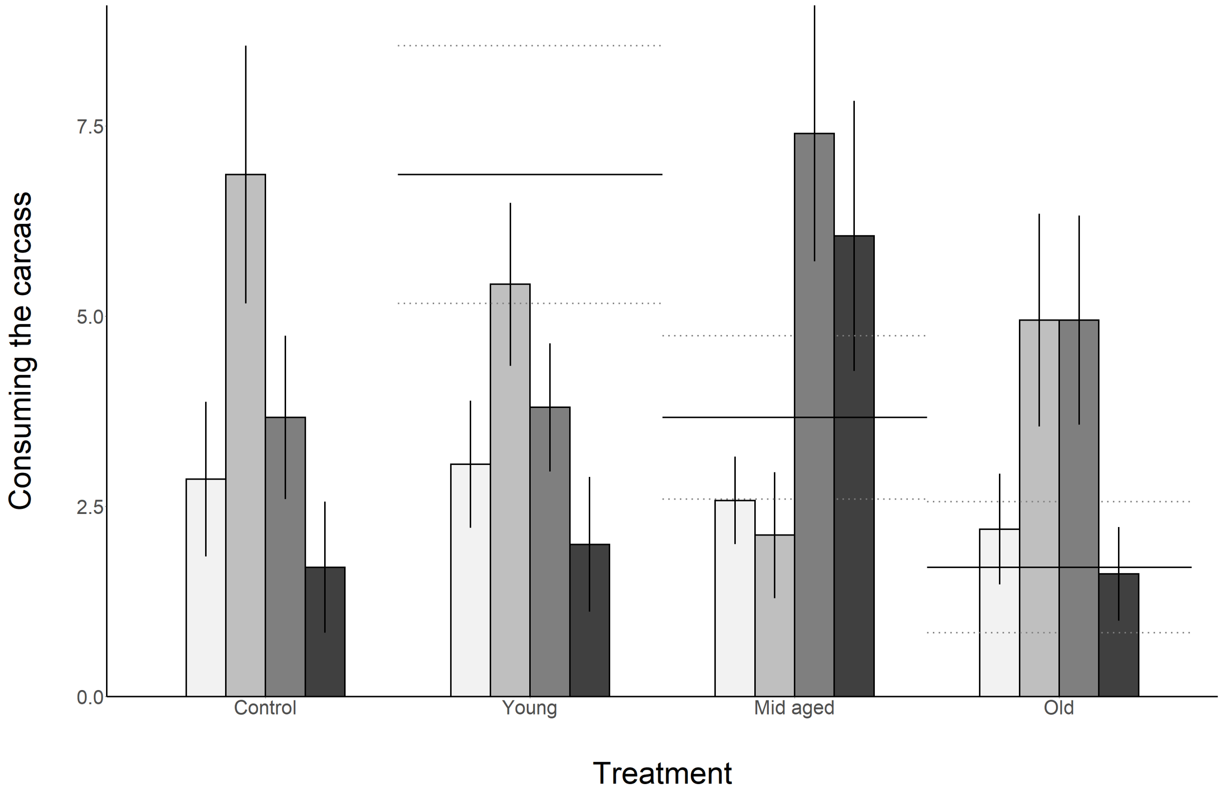

Fig. S1.3. Mean (± standard error) of the counts of occurences the female burying beetles *Nicrophorus vespilloides* consuming the carcass during the 30 min behaviour observations conducted in 24 hour interval. Dark line over each experimental treatment corresponds to the behaviour levels of the treatment with same-aged larvae in the control treatment and its standard error (dotted lines).

Table S1.4. Occurences of the female burying beetles *Nicrophorus vespilloides* engaged in non-parental behaviours during the 30 min behaviour observations. For each factor on the treatment level, there is information on parameter estimate (Par), standard error (SE), test statistic (Z value), and the P value (P). Analyses were conducted with generalized linear mixed effects models (glmmADMB) with experimental block and the identity of the female assigned as random factors.

Factor	Par	SE	Z value	P
Young	-0.252	0.317	-0.796	0.426
Mid-aged	-0.769	0.348	-2.210	0.027
Old	-0.912	0.373	-2.442	0.015
Time(25)	0.282	0.358	0.788	0.431
Time(49)	0.266	0.358	0.742	0.458
Time(73)	-0.116	0.365	-0.319	0.750
Young:Time(25)	0.162	0.449	0.362	0.718
Mid-aged:Time(25)	0.919	0.488	1.885	0.059
Old:Time(25)	0.860	0.523	1.644	0.100
Young:Time(49)	0.356	0.449	0.793	0.428
Mid-aged:Time(49)	0.607	0.502	1.208	0.227
Old:Time(49)	0.733	0.527	1.390	0.165
Young:Time(73)	-0.018	0.474	-0.039	0.969
Mid-aged:Time(73)	1.002	0.510	1.964	0.050
Old:Time(73)	1.278	0.531	2.405	0.016

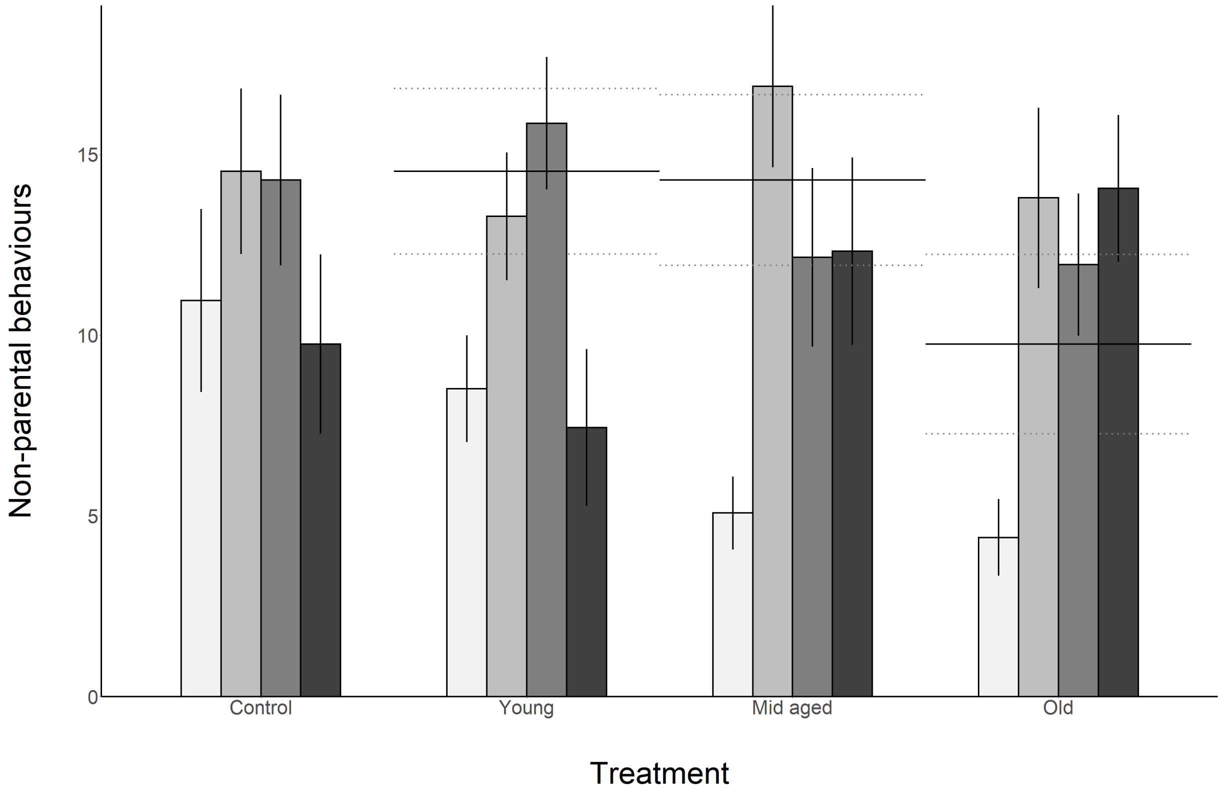

Fig. S1.4. Mean (± standard error) of the counts of occurences the female burying beetles *Nicrophorus vespilloides* engaged in non-parental behaviours during the 30 min behaviour observations conducted in 24 hour interval. Dark line over each experimental treatment corresponds to the behaviour levels of the treatment with same-aged larvae in the control treatment and its standard error (dotted lines).

Table S1.5. Occurences of the female burying beetles *Nicrophorus vespilloides* being away from the carcass during the 30 min behaviour observations. For each factor on the treatment level, there is information on parameter estimate (Par), standard error (SE), test statistic (Z value), and the P value (P). Analyses were conducted with generalized linear mixed effects models (glmmADMB) with experimental block and the identity of the female assigned as random factors.

Factor	Par	SE	Z value	P
Young	0.012	0.777	0.016	0.987
Mid-aged	-0.027	0.842	-0.032	0.974
Old	0.144	0.897	0.161	0.872
Time(25)	-2.120	0.906	-2.339	0.019
Time(49)	-0.744	0.889	-0.837	0.402
Time(73)	0.272	0.896	0.304	0.761
Young:Time(25)	1.107	1.126	0.984	0.325
Mid-aged:Time(25)	1.565	1.213	1.290	0.197
Old:Time(25)	1.300	1.293	1.005	0.315
Young:Time(49)	-0.053	1.114	-0.048	0.962
Mid-aged:Time(49)	-1.077	1.244	-0.865	0.387
Old:Time(49)	-1.110	1.298	-0.855	0.393
Young:Time(73)	0.361	1.159	0.312	0.755
Mid-aged:Time(73)	-0.734	1.248	-0.588	0.557
Old:Time(73)	-0.916	1.295	-0.708	0.479

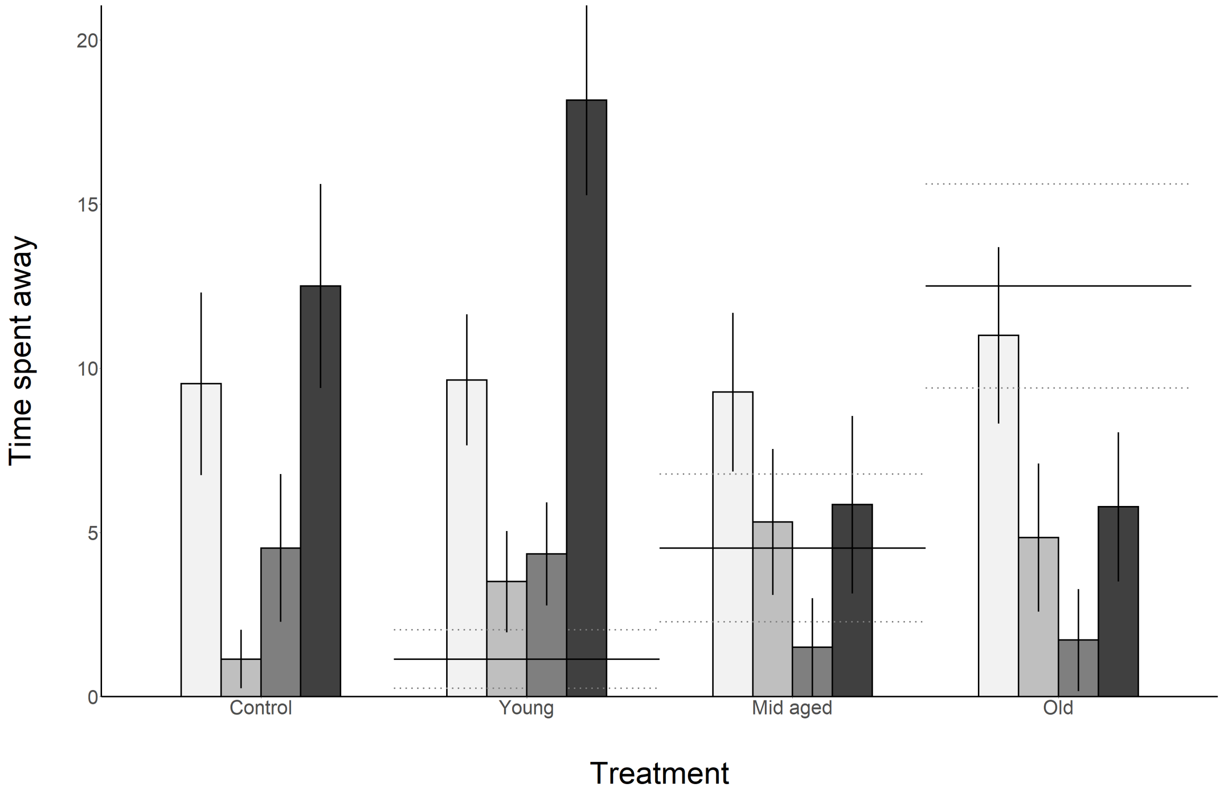

Fig. S1.5. Mean (± standard error) of the counts of occurences the female burying beetles *Nicrophorus vespilloides* being away from the carcass during the 30 min behaviour observations conducted in 24 hour interval. Dark line over each experimental treatment corresponds to the behaviour levels of the treatment with same-aged larvae in the control treatment and its standard error (dotted lines).

Table S1.6. Occurences of the female burying beetles *Nicrophorus vespilloides* being within a pronotum's distance away from (i.e. being in the proximity of) the larvae during the 30 min behaviour observations. For each factor on the treatment level, there is information on parameter estimate (Par), standard error (SE), test statistic (Z value), and the *P* value (*P*). Analyses were conducted with generalized linear mixed effects models (glmmADMB) with experimental block and the identity of the female assigned as random factors.

Factor	Par	SE	Z value	*P*
Young	0.223	0.267	0.836	0.403
Mid-aged	0.446	0.270	1.652	0.099
Old	0.261	0.283	0.924	0.356
Time(25)	0.421	0.282	1.494	0.135
Time(49)	0.024	0.294	0.081	0.935
Time(73)	0.109	0.352	0.311	0.756
Young:Time(25)	-0.210	0.363	-0.579	0.563
Mid-aged:Time(25)	-0.807	0.435	-1.857	0.063
Old:Time(25)	-0.467	0.406	-1.149	0.251
Young:Time(49)	0.119	0.375	0.318	0.751
Mid-aged:Time(49)	0.179	0.394	0.455	0.649
Old:Time(49)	-0.242	0.397	-0.610	0.542
Young:Time(73)	0.003	0.488	0.006	0.995
Mid-aged:Time(73)	0.008	0.452	0.017	0.986
Old:Time(73)	-0.700	0.450	-1.555	0.120

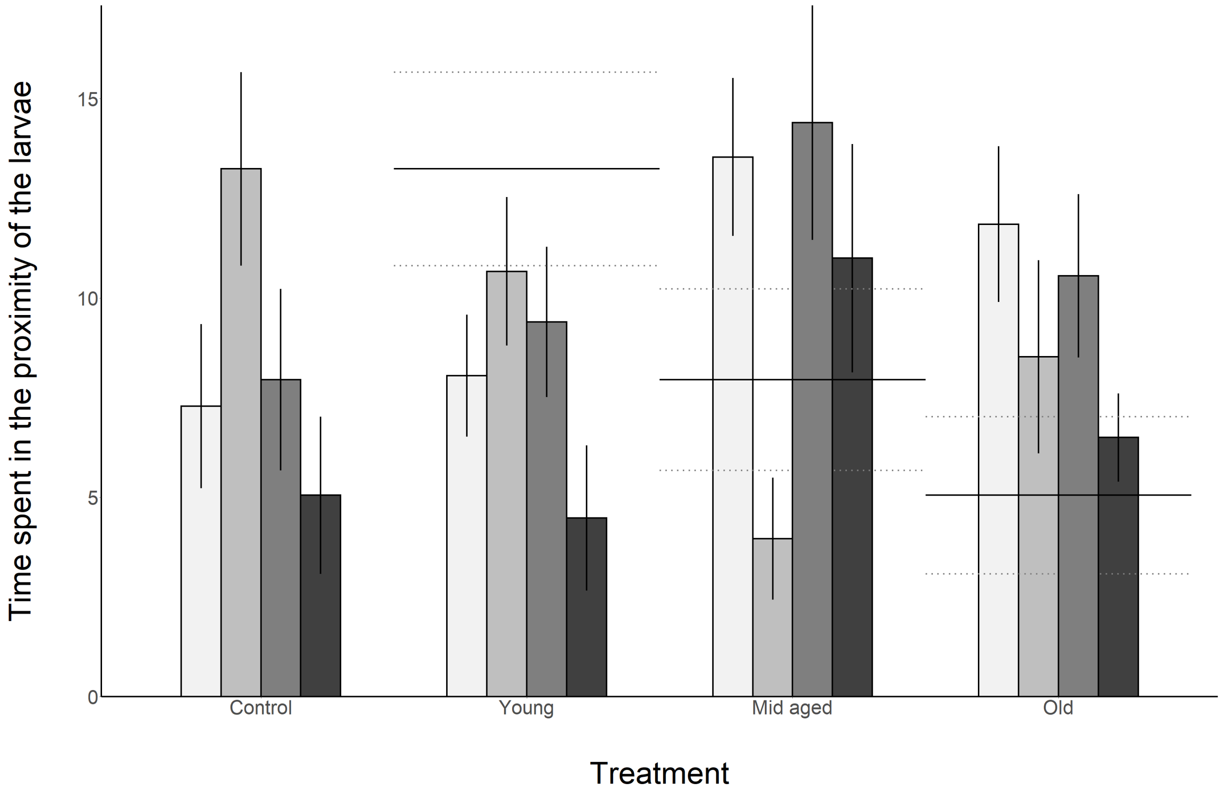

Fig. S1.6. Mean (± standard error) of the counts of occurences the female burying beetles *Nicrophorus vespilloides* being within a pronotum's distance away from (i.e. being in the proximity of) the larvae during the 30 min behaviour observations conducted in 24 hour interval. Dark line over each experimental treatment corresponds to the behaviour levels of the treatment with same-aged larvae in the control treatment and its standard error (dotted lines).

References

[1] R Core Team 2020 R: A language and environment for statistical computing, Vienna, Austria. URL http://www.R-project.org/.

[2] Fournier, D. A., Skaug, H.J., Ancheta, J., Ianelli, J., Magnusson, A., Maunder, M. N., Nielsen, A. & Sibert, J. 2012 AD Model Builder: using automatic differentiation for statistical inference of highly parameterized complex nonlinear models. *Optim. Methods Softw.* **27**, 233-249.

[3] Skaug, H., Fournier, D., Bolker, B.M., Magnuson, A. & Nielsen, Al. 2014 Generalized Linear Mixed Models using AD Model Builder. R package version 0.8.0. URL http://glmmadmb.r-forge.r-project.org/
